# Linking Biodiversity Conservation and Livelihoods in India

**DOI:** 10.1371/journal.pbio.0030394

**Published:** 2005-11-15

**Authors:** Kartik Shanker, Ankila Hiremath, Kamal Bawa

## Abstract

The Ashoka Trust for Research in Ecology and the Environment was established in 1996 to curtail the rapid loss of India's biological resources and natural ecosystems.

In a country like India, millions of people rely on products from natural ecosystems to sustain their livelihood. Natural ecosystems provide clean water from watersheds, retention of soil and soil fertility, sequestration of carbon, as well as pollinators and natural predators of pests. For these reasons, rapid, often irreversible, loss of species and ecosystems is of more than just academic concern. Indeed, our understanding of biodiversity in natural ecosystems remains so woefully inadequate that we are unable to fully comprehend the consequences of its loss. With impending climate change and increasing spread of invasive species, the biodiversity crisis is likely to get worse, with far-reaching effects on human societies.

To meet these challenges, we need institutions and scholars that can generate new knowledge, and apply it to resolve our most pressing environmental issues. The Ashoka Trust for Research in Ecology and the Environment (ATREE) (http://www.atree.org) was established in 1996 to curtail the rapid loss of India's biological resources and natural ecosystems, and to address the environmental, social, and economic dimensions of this decline. We highlight below two examples of ATREE's work from very different ecosystems and regions.

## Tribals and Non-Timber Forest Products in the Western Ghats

The Biligiri Rangaswamy Temple Wildlife Sanctuary (BRT), a 540-square-kilometer protected area, forms a part of India's Western Ghats, one of the global hotspots of biodiversity. The area has traditionally been inhabited by an indigenous community, the Soligas, and is also a habitat for a number of endangered plants and animals. Soligas have harvested forest products for centuries for their own use and more recently for markets. The interrelated issues of livelihood enhancement of the Soligas and biodiversity conservation have been at the heart of ATREE's work in BRT for close to a decade—along with a partner non-governmental organization, the Vivekananda Girijana Kalyana Kendra; a local community organization, the Soliga Abhivrudhi Sangha; and the Karnataka Forest Department [1]. A detailed understanding of the drivers that cause forest loss and degradation is the first step toward its preservation. [Fig pbio-0030394-g001]

**Figure pbio-0030394-g001:**
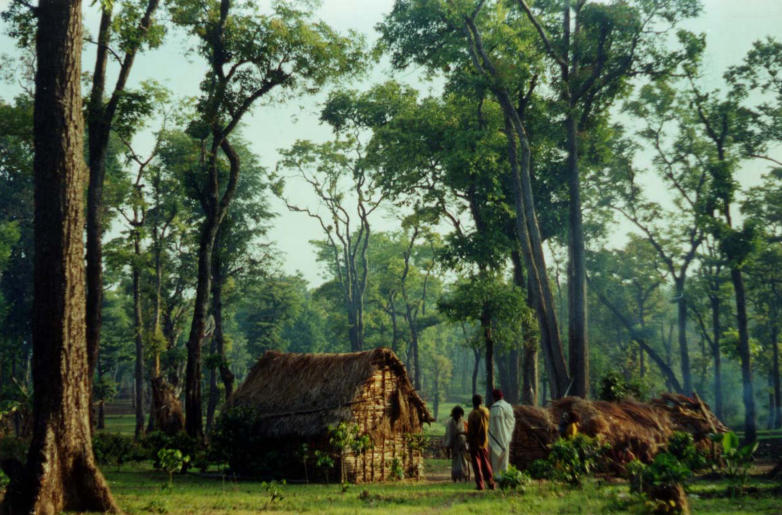
The forestland of India's Western Ghats is a target for conservation, which must involve local communities (Photo: Smitha Krishnan)

We have used demographic models to analyze changes in population structure of nelli (Phyllanthus emblica and Phyllanthus indofischeri), one of the most important NTFPs in BRT. Nelli is an edible fruit high in vitamin C, extracts from which are key ingredients in traditional Indian medicine, and in cosmetics. Our results indicate that population growth rates, on average, are close to rates that would allow full replacement of individuals. Moreover, it is not harvest, per se, but rather the method of harvest involved (e.g., whether or not it involves the lopping off of branches or cutting of small trees) and the spread of Loranthus, a plant parasite that infests mature trees, which affects population growth [2]. Management efforts, therefore, need to focus on control of the parasite, and on the use of nondestructive harvest techniques.

Discussion of harvest techniques and parasite removal form part of the annual participatory monitoring meetings with Soliga harvesters. These participatory monitoring meetings also focus on the temporal and spatial patterns in availability of various NTFPs, which are then used to guide harvest decisions. Information collected as part of this participatory monitoring program corroborate the results of scientific monitoring, namely, that populations of nelli, and also those of other important NTFP species, such as the Asian honeybee (Apis dorsata), a source of honey, have remained relatively stable over the last ten years.

It is essential that the benefits accruing to harvesters be maximized for their continued participation in management and conservation. A large part of our effort to strengthen existing institutions has, therefore, been directed toward reform of the Large-Scale Adivasi (tribal) Multi-Purpose Society, a government-established cooperative society, and a key element in the success of efforts at forest conservation. Non-timber products harvested from the forest can only be sold to the Large-Scale Adivasi Multi-Purpose Society. ATREE is also working with Soliga farmers to increase agricultural productivity, enhance on-farm diversity, and improve soil and water conservation. It is hoped that through simple agricultural interventions we can achieve a greater on-farm contribution toward subsistence and cash needs, thereby reducing the extent of dependence on NTFP.

BRT is the only forest area in India where production and extraction of NTFPs are being monitored, and where the local community is involved in such monitoring. In a recent meeting with the Forest Department, a committee comprising members of the Soliga community, Vivekananda Girijana Kalyana Kendra, and ATREE was proposed to provide suggestions to the Forest Department on management of the protected area. If formalized, this would make BRT the first protected area to have such a three-way collaboration between managers, the local community, and researchers, and would be a model for other protected areas in the country.

## Protecting Turtles with Fisherfolk

On the other side of the country, on the coast of Orissa, are the nesting grounds of olive ridley sea turtles (Lepidochelys olivacea). This is one of three rookeries worldwide where arribadas, the synchronous mass nesting of thousands of ridley turtles, occurs. Genetic studies demonstrate that this is a unique population, which may be ancestral to olive ridleys in other ocean basins [3]. In the last decade, however, 10,000 turtles have been counted dead on the Orissa coast each year due to fishery-related activities. And many more are likely to have died, since not all turtles killed in nets are washed ashore [4].

Research indicates that olive ridleys remain in small offshore congregations during the breeding season, and are not diffusely distributed along the entire coast. Discrete reproductive patches off the mass nesting beaches are usually no more than 50 square kilometers; however, the location of these patches may vary over time [5]. Thus, the creation of sanctuaries or protected areas with fixed boundaries may not be effective. Instead, conservationists have focused on the enforcement of laws, including existing fishery regulations such as the 1983 Orissa Marine Fisheries Regulation Act, which stipulates that mechanized fishing is prohibited within five or ten kilometers of the coast, depending on boat size.

Despite the investment of large amounts of effort and funds by the government and civil society groups to patrol nearshore waters, trawlers continue to fish illegally in nearshore waters, causing continuing mortality of olive ridleys. Rather, the anti-trawler programs, coupled with the media coverage, have severely polarized fishing communities and conservation groups. Even traditional fish-worker associations in Orissa joined in protests with the trawler owners, since they perceived turtle conservation as being anti-people, even though most of the Orissa Marine Fisheries Regulation Act regulations were designed to protect traditional fishing rights rather than turtles.

In fact, if fishery laws had been enforced for the reasons that they were originally instituted, namely, to protect traditional fisherfolk and their livelihoods, it is likely that their implementation would have received far wider support. And as a result, sea turtles would then have been protected from mechanized fishing. To achieve this goal, the Coastal and Marine Programme at ATREE created and facilitated a common platform for sea turtle conservation in Orissa. In December 2004, ATREE organized a meeting in Bhubaneshwar that was attended by key fish-worker organizations (Orissa Traditional Fish Workers' Union and United Artists Association), local community organizations, and non-governmental organizations such as Project Swarajya, Wildlife Society of Orissa, Worldwide Fund for Nature, Greenpeace, and others. The group named itself the Orissa Marine Resources Conservation Consortium, and has been working together to achieve common marine conservation goals.

The Orissa Marine Resources Conservation Consortium has held numerous follow-up meetings in 2005. The activities identified to meet these common goals included meetings for fisherfolk representatives and other stakeholders on fisheries management and turtle conservation legislation in April 2005. ATREE has produced illustrative local language booklets on fisheries conservation and turtle protection measures in Orissa to help local communities understand their rights and regulations. Booklets, along with other visual aids such as posters illustrating fishing regulations, have been distributed to various stakeholders at the mass nesting beaches. The Orissa Marine Resources Conservation Consortium plans to promote community-based marine conservation practices and appropriate environmentally sustainable coastal development, addressing the issues of marine biodiversity and resource use.

## The Big Picture

With its headquarters in Bangalore, ATREE also has offices in Delhi, and northeast India, where its programs are directed toward conservation of the eastern Himalayan region. ATREE's current activities are grouped under research and action, education, and outreach. Specifically, ATREE uses interdisciplinary approaches to (1) generate knowledge that fosters conservation and judicious management of biodiversity, (2) provide the best scientific information to policymakers, (3) design management systems that emphasize decentralization, fairness, and equity in the use of resources by civil society, (4) organize and disseminate information for conservation and sustainable use of biodiversity, and (5) train a new generation of leaders to meet current challenges in biodiversity conservation and environmental protection.

The various activities are organized under three centers: the Center for Conservation Science; the Center for Conservation, Governance, and Policy; and the Center for Ecoinformatics (http://www.ecoinfoindia.org). Most of the existing staff work in diverse areas of natural and social sciences including biodiversity characterization, forest ecology, taxonomy, conservation genetics, landscape ecology, hydrology, environmental sociology, and ecological economics, in the Center for Conservation Science. All three centers are, or would be, engaged in education and outreach activities designed to build the capacity of academic, governmental, and non-governmental organizations to meet the growing list of contemporary environmental challenges.

## Abbreviations

ATREE: Ashoka Trust for Research in Ecology and the Environment
BRT: Biligiri Rangaswamy Temple Wildlife Sanctuary
NTFP: non-timber forest product
